# Design and evaluation of FEBS Education and Training Academy module – Integrating generative artificial intelligence into life‐science education

**DOI:** 10.1002/2211-5463.70359

**Published:** 2026-10-26

**Authors:** Zvonimir Mlinarić, Ali Burak Özkaya

**Affiliations:** ^1^ Department of Pharmaceutical Analysis, Faculty of Pharmacy and Biochemistry University of Zagreb Croatia; ^2^ Faculty of Medicine İzmir University of Economics Türkiye

**Keywords:** academic integrity, assessment, faculty development, generative artificial intelligence, higher education, life‐science education

## Abstract

Generative artificial intelligence (AI) is now embedded in higher education. Yet educators often adopt it more slowly and cautiously than students, and generic guidance rarely translates into changes in teaching or assessment practice. We designed a short, hands‐on, pedagogy‐first workshop in which life‐science educators work with their own course materials, intended to increase confidence in pedagogically informed AI use and prompt concrete changes to course design, teaching and assessment. However, this is an exploratory feasibility and specification report; therefore, the evaluation was not designed to test that intention. Here we describe the design of a transferable three‐session FEBS Education and Training Academy module, organised around the PICRAT (Passive‐Interactive‐Creative/Replace‐Amplify‐Transform) framework, a four‐element prompt structure and studio‐style application to participants' own teaching artefacts, and evaluate it using matched pre/post surveys, anonymous after‐session feedback and an online follow‐up meeting. Thirty‐four educators from 14 countries attended; 21 completed the pre‐workshop and 13 the post‐workshop survey (12 matched). Among the 12 respondents who completed both surveys, the proportion reporting high confidence rose by the same margin, 4 of 12, in each of the five practical domains. Ten of 13 follow‐up respondents reported implementing at least one idea and while views of AI as beneficial were already positive and changed little, agreement that AI poses significant risks to academic integrity fell. These participant‐reported, uncontrolled findings should be read as evidence of feasibility and perceived usefulness rather than causal impact.

## Graphical abstract

Life‐science educators from 14 countries redesigned their own course materials with generative AI in a three‐session, pedagogy‐first FEBS Education and Training Academy workshop built on PICRAT. Among 12 matched respondents, four more reported high confidence in each of five practical domains, and 10 of 13 reported implementing at least one idea. Session plans, prompts and instruments are provided for reuse.

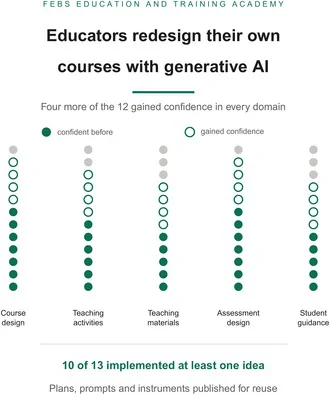

**Abbreviations**
AI: artificial intelligence
CHERRIES: Checklist for Reporting Results of Internet E‐Surveys
ETC: Education and Training Conference
FEBS: Federation of European Biochemical Societies
ILO: intended learning outcome
PICRAT: passive‐interactive‐creative/replace‐amplify‐transform
SRQR: Standards for Reporting Qualitative Research
TPACK: technological pedagogical content knowledge

Generative artificial intelligence (AI) has rapidly become part of everyday higher education practice. Student use is now widespread, including for assessed work, whereas educator use is substantial but often more cautious, uneven and concentrated in teaching‐preparation tasks rather than assessment‐critical decisions [1, 2]. This creates a practical challenge for universities: students and educators frequently engage with the same technologies at different speeds, with different levels of guidance, confidence and institutional support.

The key educational question is therefore no longer whether AI should be present in higher education, but how it should be integrated. Generative AI can support teaching and learning by helping educators review alignment among learning outcomes, activities, materials and assessment; generate examples; design activities; provide feedback; or explore alternative assessment formats. At the same time, it can allow students to produce polished outputs without engaging in the intended learning process [3]. This distinction is particularly important in life‐science education, where students must develop conceptual understanding, scientific reasoning, accuracy in interpreting representations and professional judgement.

Assessment has become one of the most immediate pressure points. Responses based mainly on prohibition, policing, or AI detection are limited by concerns about accuracy, fairness, and practicality [4, 5]. More sustainable approaches require assessment designs that acknowledge possible AI use, give students transparent guidance on appropriate and inappropriate use, and focus more explicitly on process, reasoning, documentation, reflection and student responsibility [6]. The shift has been articulated in the assessment literature as a move from assessment security towards assessment design and transparency and operationalised in approaches that deliberately separate tasks in which AI use is permitted and disclosed from those in which it is not [7, 8]. In this paper, we use the term AI‐aware assessment as a convenient label for this established family of approaches rather than as a new construct. The aim is not simply to make assessment ‘AI‐proof’, but to make it AI‐aware and educationally meaningful.

Educators therefore need faculty development that goes beyond lists of tools or generic policy statements. They need opportunities to work with their own courses, materials and assessment tasks, to test AI outputs, to discuss limitations and to decide where AI can support existing educational goals. Frameworks such as PICRAT (Passive‐Interactive‐Creative/Replace‐Amplify‐Transform) are useful in this context because they shift attention from tool adoption to pedagogical purpose: whether technology merely replaces existing practice, amplifies it or transforms it [9]. We selected PICRAT in preference to broader technology‐integration frameworks such as TPACK [10] because its instructor‐side Replace‐Amplify‐Transform axis maps directly onto the concrete artefacts (course outcomes, activities, materials and assessment tasks) that participants would be revising during the studio sessions. Accordingly, our workshop was designed as a pedagogy‐first activity in which AI tools were introduced in relation to course design, learning activities, materials and assessment tasks, rather than as tools to be adopted for their own sake.

AI integration also requires continuing peer exchange, because tools change quickly, institutional policies vary, and implementation often reveals new practical problems, including hallucinations, inaccurate scientific visuals, data‐privacy concerns, unequal access and uncertainty about appropriate student use. For this reason, AI‐related faculty development should be understood not only as individual skills training but also as a basis for local and disciplinary communities of practice [11, 12]. Emerging competency frameworks for teachers' use of AI [13] and analyses of generative‐AI professional‐development needs among teachers [14] similarly emphasise that effective support must combine practical skills, ethical judgement, and sustained collegial exchange rather than one‐off tool demonstrations.

Comparable faculty‐development interventions have begun to be evaluated. A workshop series for medical faculty framed around TPACK reported gains in self‐reported confidence and in initial strategies for responsible AI integration, with the same limitations of small sample size, single setting and reliance on self‐report [15]. A pre/post evaluation of an introductory generative‐AI course for higher‐education staff reported increased intention to use these tools [16]. What has not been established is whether a short, discipline‐specific activity in which participants work throughout on their own teaching materials produces a comparable effect in the molecular life sciences, or whether such an activity can be specified in sufficient detail for others to re‐run it.

We therefore developed and evaluated a transferable workshop model within the FEBS Education and Training Academy, an initiative of the FEBS Education and Training Committee that supports professional development, educational exchange and community‐building in molecular life sciences education [17]. The workshop was designed with two intended outcomes: that a short, hands‐on, pedagogy‐first activity in which life‐science educators work with their *own* teaching materials would (a) increase confidence in pedagogically informed AI use across course design, teaching activities, materials, assessment and student guidance, and (b) prompt concrete, actionable changes that participants would begin to implement in their own contexts. The evaluation reported here was not designed to test those expectations against a comparable condition. It is presented as an exploratory feasibility and specification report, intended to establish whether the model is workable and reusable and to generate hypotheses for future controlled work. In this paper, we describe the design of the FEBS Education and Training Academy workshop ‘Integrating AI into Education: Enhancing Course Design, Teaching, and Assessment’ and report a mixed‐methods evaluation of its feasibility, perceived usefulness, participant‐reported outcomes and early implementation. Accordingly, this paper reports a specification rather than a single‐event report so that FEBS Education and Training Academy and comparable disciplinary networks can re‐run and adapt for AI faculty development in the molecular life sciences.

## Materials and methods

### Study design and evaluation questions

This was a single‐cohort, mixed‐methods evaluation of a faculty‐development workshop. Quantitative data were collected through matched pre‐ and post‐workshop surveys and analysed descriptively; qualitative data were collected through anonymous after‐session ‘one‐minute papers’ and an online follow‐up meeting and analysed using a descriptive thematic/content‐analysis approach. The qualitative analysis and its reporting were informed by the Standards for Reporting Qualitative Research (SRQR) [18], while the design and reporting of the survey followed the Checklist for Reporting Results of Internet E‐Surveys (CHERRIES) [19]. The evaluation was exploratory and was not designed to provide controlled or causal evidence of effectiveness. The evaluation addressed four questions: whether the workshop could be delivered as specified and was perceived as useful; whether participants' reported confidence and attitudes changed after the workshop; whether participants reported implementing workshop ideas in their own teaching; and which needs for further support remained.

### Workshop context and participants

The workshop ‘Integrating AI into Education: Enhancing Course Design, Teaching, and Assessment’ was delivered during FEBS ETC 2026, held in Kuşadaşı, Türkiye, from 25 to 28 March 2026. The workshop itself took place between 26 and 28 March 2026, with one 2‐h session delivered on each day. It was designed for academics and course coordinators involved in life‐science education and was delivered as an in‐person, laptop‐based FEBS Education and Training Academy educational activity. Thirty‐four participants from 14 countries attended the workshop and worked in six groups. The groups were self‐selected and remained stable across the three sessions. The workshop comprised three face‐to‐face sessions and a planned post‐workshop follow‐up component. The follow‐up component consisted of a post‐workshop survey collected in April and May 2026 and an online discussion meeting held on 6 June 2026 via Microsoft Teams.

### Workshop aims and learning goals

The workshop aimed to support educators in making pedagogically informed decisions about the use of generative AI in teaching, learning and assessment. The focus was on helping participants evaluate when AI may support learning, when it may be inappropriate and how its use can be made transparent, ethical and educationally meaningful.

The learning goals were for participants to: describe the main functions, benefits, and limitations of generative AI tools relevant to teaching and learning; identify ethical, privacy‐related, and bias‐related considerations; apply basic prompting strategies to obtain useful feedback or suggestions; revise course elements such as outcomes, activities, materials, or assessments using AI‐supported feedback and their own judgement; and outline transparent guidance for appropriate AI use in their own courses.

### Workshop design: session sequence, PICRAT framework and prompting structure

The workshop was designed as a hands‐on faculty‐development activity rather than a tool demonstration. Each session combined short conceptual briefings, live demonstrations, group discussion, and studio‐style application. A central design principle was that participants worked with their own teaching materials rather than generic examples. Before the workshop, participants were asked by e‐mail to bring materials from one of their own courses, such as syllabi, course outcomes, weekly plans, lecture slides, learning activities, assignments, rubrics, or assessment questions (Fig. 1).

**Fig. 1:**
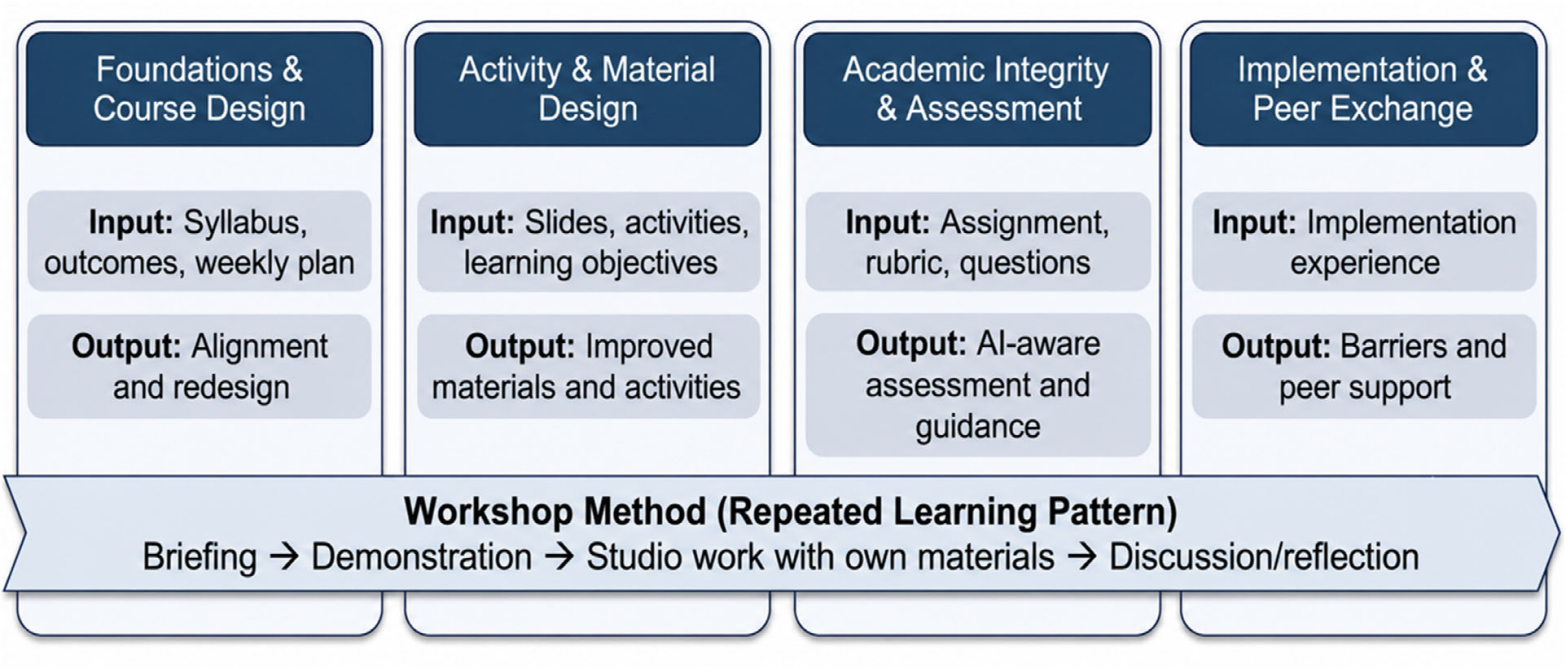
Structure of the FEBS Education and Training Academy workshop. The workshop consisted of three 2‐h face‐to‐face sessions followed by a post‐workshop follow‐up component. Each session used a repeated learning pattern of briefing, demonstration, studio work with participants' own teaching materials, and discussion/reflection. The sequence moved from course design to activity and material design, and then to academic integrity and assessment, followed by implementation‐focused peer exchange.

The three‐session sequence moves from course‐level design toward activity/material design and then assessment. Session 1 introduced the foundations of generative AI and applied these concepts to course design, including course outcomes, syllabus structure, weekly plans, alignment and assessment strategies. Session 2 focused on teaching activities and learning materials, including active‐learning design, slide review, examples, cases, study notes, visuals and alignment with learning goals. Session 3 focused on academic integrity and assessment, including the limitations of banning or detecting AI use, AI‐aware assessment design, student guidance, process‐oriented assessment, rubrics and assessment redesign (Fig. 1). Session‐by‐session plans with timings, and the instructions sent to participants before the workshop are given in Doc S1.

The educational design was informed by the PICRAT framework, particularly the instructor‐side distinction between using technology to replace, amplify, or transform existing practice. Participants were encouraged to consider how AI could amplify or transform course design, teaching activities, learning materials, and assessment practices, rather than merely replacing existing tasks. Both axes of the framework were introduced in the Session 1 foundation briefing, and the educator‐side axis was presented again in every subsequent briefing. In Session 1, it was shown a second time immediately after a live demonstration in which a third‐year Biochemistry I syllabus was reviewed with a generative AI tool, and before the studio task (Doc S1).

Prompting was treated as a practical design skill. Participants were introduced to a simple prompt structure based on four elements: context, task, constraints and expected output. Example prompts were provided for common educational tasks, including syllabus review, alignment checking, activity redesign, generation of teaching materials, assessment analysis, rubric development and AI‐aware assessment design. The complete set is given in Doc S2. Participants then adapted these prompts to their own courses, institutional constraints, student level and educational goals.

The studio tasks followed a common pattern: participants selected a real educational artefact from their own teaching, used an AI tool to review, critique, revise or redesign it, and then discussed the usefulness, limitations and risks of the output with peers and facilitators. Participants were not asked to accept AI‐generated outputs directly; the emphasis was on instructor judgement, quality control, disciplinary accuracy and alignment with intended learning goals.

The post‐workshop follow‐up component was included to support reflection and implementation after participants returned to their own institutions. Its purpose was to discuss what participants had tried, what barriers they encountered, what additional support would have been useful and what they planned to do next.

### Data collection: surveys, one‐minute papers and follow‐up meeting

Workshop evaluation data were collected through four sources.

First, a pre‐workshop survey was distributed on 18 March 2026 and remained open until the start of the workshop on 26 March 2026. The pre‐workshop survey was completed by 21 participants. It collected information on participants' roles, teaching experience, prior AI use, frequency of AI use, previous modification of teaching or assessment practices because of AI, confidence in AI‐supported educational tasks, views and concerns regarding AI in education, perceived institutional guidance and expected outcomes from the workshop. Responses were name‐linked to allow pre/post matching. The full wording of every survey item, including the five confidence items and the four attitude items, together with the 5‐point response anchors and the midpoint label, is given in Doc S3. The instruments were developed by the two facilitators for this evaluation and were not based on a previously validated scale. One pre‐workshop confidence item, on critically evaluating AI‐generated outputs, was not repeated in the post‐workshop survey and is not analysed further.

Second, one‐minute paper activities were completed anonymously after each face‐to‐face session and collected digitally using Google Forms. Each one‐minute paper asked participants to identify one change they planned to make after the session and one remaining uncertainty or challenge. The wording differed by session to match its topic and is given in Doc S4. Responses were collected from 17 participants after Session 1, 16 after Session 2, and 18 after Session 3.

Third, a post‐workshop survey was collected during April and May 2026. This survey was completed by 13 participants and functioned as the post‐workshop/follow‐up survey. It repeated several pre‐workshop items on confidence, attitudes and concerns, and also asked participants whether they had implemented at least one idea from the workshop. The implementation item used the response options ‘Yes’, ‘Yes, but not as much as I wanted to’, ‘Not yet, but planning to’ and ‘No’. The survey also asked whether participants planned to attend the online follow‐up meeting and included open‐text questions about implementation, planned implementation and additional comments. Responses were name‐linked to allow matched analysis with the pre‐workshop survey.

Fourth, an online follow‐up meeting was held on 6 June 2026 via Microsoft Teams. The meeting was structured around four questions circulated in advance: what participants had implemented and how it went; what challenges they encountered; what additional support, knowledge or resources would have helped; and what they planned to try next (Doc S4). The meeting was attended by four participants and two facilitators. Meeting notes were used descriptively to contextualise implementation, barriers and support needs.

### Quantitative data analysis: changes in confidence and attitudes, and implementation

Survey responses were analysed descriptively. Participant characteristics, prior AI use, frequency of AI use, previous modification of practice, implementation status and follow‐up attendance intention were summarised using counts and percentages. Item‐level missing responses were handled using the number of valid responses for each item as the denominator.

Two baseline AI‐use variables were distinguished. ‘Prior AI use’ referred to participants' stage of familiarity or adoption of AI tools for teaching‐related tasks, ranging from not familiar to regular use. ‘Frequency of AI use’ referred to how often participants reported using AI tools for teaching‐related tasks, ranging from never to daily.

Likert‐type items were coded from 1 to 5, with higher scores indicating stronger agreement or higher confidence. For group‐level pre/post comparisons, repeated items present in both the pre‐workshop and post/follow‐up surveys were summarised as the number and percentage of respondents selecting 4 or 5. For confidence items, scores of 4–5 were interpreted as higher confidence; for attitude items, scores of 4–5 were interpreted as agreement with the statement. One of the five repeated practical items, on providing students with guidance on AI use, describes current practice rather than confidence, and its wording was broadened between the two surveys from the course brought to the workshop to all the courses a participant teaches (Doc S3). It is reported with the confidence items for brevity, but it should be read as reported practice, and its pre/post comparison is not strictly like for like. No inferential statistical tests were conducted for group‐level pre/post comparisons because the evaluation was exploratory, the sample size was small, and the pre‐ and post‐workshop respondent groups were not completely identical.

For within‐participant comparison, an exploratory matched‐pair analysis was conducted for the 12 respondents who completed both surveys and could be matched (12 of the 13 post‐workshop respondents; one post‐workshop response could not be linked to a pre‐workshop response). Matching was based on participant names, with spelling and diacritic differences normalised where necessary. For each repeated item, the direction of change was coded as a higher post‐workshop score, an unchanged score or a lower post‐workshop score. These categories were interpreted as the direction of change on the response scale rather than automatically as improvement or deterioration. Because the 12 matched respondents constitute 12 of the 13 post‐workshop respondents, the group‐level and within‐participant analyses draw on almost the same data and are not treated as mutually corroborating; to provide a like‐for‐like comparison, the proportion selecting 4 or 5 is additionally reported for these 12 participants at both time points. Direction‐of‐change tallies were tested using exact two‐sided sign tests. Tied pairs were excluded, and pairs in which either the pre‐ or the post‐workshop response to that item was missing were also excluded, so the number of evaluable pairs for an item is 12 minus its tied and incomplete pairs. No pair was imputed. Analyses were performed in r version 4.6.0 (R Foundation for Statistical Computing, Vienna, Austria), using binom.test for the exact sign tests and cor.test with method = ‘spearman’ for the rank correlations. No correction for multiple comparisons was applied, and these tests are reported for transparency rather than as confirmatory analyses.

Exploratory ordinal association analyses were conducted at baseline using Spearman's rank correlation. These analyses examined associations between years of teaching experience and two ordinal AI‐use variables: prior AI use for teaching‐related tasks and frequency of AI use for teaching‐related tasks. Teaching experience, prior AI use and frequency of AI use were coded as ordered categories, and Spearman's *ρ* is reported with the sample size. *P* values are not reported for these associations: the variables are ordinal with a small sample size and a high proportion of tied ranks, which are conditions under which *P* values from the normal approximation are unreliable. These analyses are treated as descriptive and hypothesis‐generating only. Because prior AI use and frequency of AI use measure closely related constructs, the two analyses should be read as a single association expressed on two scales rather than as independent findings.

### Qualitative data analysis: expectations, planned changes and remaining needs

Open‐text responses were analysed using a descriptive thematic/content‐analysis approach. Separate coding was applied to the pre‐workshop expected‐outcome responses, one‐minute paper planned‐change responses, one‐minute paper remaining‐uncertainty responses, post/follow‐up survey open comments and follow‐up meeting notes.

Because participants completed name‐linked surveys administered by their own facilitators, and because the same individuals designed the activity, collected the data, and coded the responses, both strands of the evaluation are susceptible to social‐desirability and demand effects [20]. No procedural safeguard against these effects, such as independent survey administration, anonymous linking codes, or coding blinded to respondent identity, was in place, and this should be weighed when interpreting every participant‐reported outcome below.

For the pre‐workshop expected‐outcome question, usable responses were defined as responses that contained interpretable content related to expected workshop outcomes; blank, unclear or non‐informative responses were excluded from thematic coding for that question. For the one‐minute papers, vague, missing or non‐substantive answers were retained as categories where relevant, because they provided information about the completeness and interpretability of session‐level responses.

Themes were developed descriptively from the response content while also considering the structure of the workshop. Because individual responses could contain more than one idea, codes were not mutually exclusive: a single response could contribute to more than one theme, and thematic counts may exceed the number of respondents for a given question or session. Percentages were calculated using the number of usable responses for the relevant dataset as the denominator.

For the planned‐change analysis, one‐minute paper responses were analysed separately for each session to identify the kinds of changes participants intended to make after that session. For the remaining‐uncertainty analysis, responses were also analysed separately by session to examine whether concerns shifted across the workshop sequence.

Several terms were standardised during coding. References to ILOs were coded as intended learning outcomes. ‘Alignment’ referred to relationships among learning outcomes, content, teaching activities, materials and assessment. ‘AI‐aware’ or ‘AI‐integrated’ assessment referred to assessment approaches that explicitly acknowledge, structure or guide AI use rather than only attempting to prohibit or detect it. Tool names such as chatgpt, claude, gemini and notebooklm were retained as participant‐reported examples and were not treated as endorsements of specific platforms.

Pre‐workshop expectation themes were compared descriptively with the pre‐planned workshop structure to examine alignment between participant needs and workshop scope. This was not treated as a measure of expectation fulfilment or satisfaction because the post/follow‐up survey did not directly ask whether participants' expectations had been met, and the workshop structure had already been designed before these survey responses were analysed.

Follow‐up meeting notes were reviewed descriptively to identify examples of implementation, barriers, tool workflows, student use concerns, support needs and community‐building challenges. Because only four participants attended the follow‐up meeting, these notes were used to contextualise and illustrate post‐workshop implementation rather than as a quantitative dataset.

### Ethical considerations

The surveys, one‐minute papers and follow‐up meeting were conducted as part of routine workshop evaluation. Pre‐ and post‐workshop survey responses were name‐linked to allow matching, whereas one‐minute paper responses were anonymous. Participants were informed at the point of consent that their pre‐ and post‐workshop responses would be linked by name, and that this linking would be performed by the workshop facilitators. Results were analysed and reported only in aggregate; individual participants are not identified, and open‐text comments are summarised or paraphrased without attribution. Participants were informed orally that anonymised, aggregated evaluation data might be used for research and publication. Pre‐ and post‐workshop responses were name‐linked solely to enable matched analysis; no individuals are identifiable in this report.

## Results

### Participant heterogeneity and baseline readiness

Of the 34 workshop participants, 21 completed the pre‐workshop survey (61.8%). Respondents represented a heterogeneous group in terms of academic role, teaching experience, previous use of AI and stage of AI‐related educational implementation (Table 1).

**Table 1:** Baseline characteristics and AI‐readiness of pre‐workshop survey respondents (*N* = 21).

Characteristic	*N* (%)
**Primary role**	
Professor/Associate Professor/Assistant Professor	12 (57.1)
Lecturer	5 (23.8)
Teaching or research assistant	3 (14.3)
Researcher with teaching responsibilities	1 (4.8)
**Years of teaching experience**	
0–5 years	2 (9.5)
6–10 years	7 (33.3)
11–20 years	7 (33.3)
> 20 years	5 (23.8)
**Prior use of AI tools for teaching‐related tasks**	
Not familiar	1 (4.8)
Aware, but not used	2 (9.5)
Used occasionally	12 (57.1)
Used regularly	6 (28.6)
**Frequency of AI use for teaching‐related tasks**	
Never	4 (19.0)
Monthly	10 (47.6)
Weekly	3 (14.3)
Several times per week	3 (14.3)
Daily	1 (4.8)
**Previous modification of teaching or assessment because of AI**	
No	0 (0.0)
No, but planning to modify	9 (42.9)
Yes, slightly	9 (42.9)
Yes, significantly	3 (14.3)
**My institution provides sufficient guidance on AI use**	
Strongly disagree	6 (28.6)
Disagree	5 (23.8)
Neither agree nor disagree	1 (4.8)
Agree	7 (33.3)
Strongly agree	2 (9.5)

Most respondents (85.7%) reported using AI tools for teaching‐related tasks either occasionally or regularly. However, routine use was less common: seven respondents (33.3%) reported using AI weekly or more frequently, whereas fourteen (66.7%) reported using it monthly or less frequently. Respondents also differed in the extent to which AI had already affected their teaching or assessment practice: nine (42.9%) had not yet modified their practice (but planned to), nine (42.9%) had made slight modifications and only three (14.3%) had made significant modifications. The two baseline AI‐use variables are not perfectly concordant: three respondents reported no prior use of AI, whereas four reported a current use frequency of never, reflecting at least one respondent who had used AI previously but was not doing so at the time of the survey.

Exploratory ordinal association analyses suggested that participants with more teaching experience tended to report lower prior AI use for teaching‐related tasks (Spearman's *ρ* = −0.49, *n* = 21) and lower frequency of AI use (*ρ* = −0.46, *n* = 21). Given the small sample, ordinal variables and high proportion of tied ranks, these associations should be interpreted descriptively rather than as formal hypothesis tests.

### Participant‐reported changes in confidence, attitudes and implementation

Participant‐reported confidence and attitudes were compared using repeated items present in both the pre‐workshop and post/follow‐up surveys. Because the pre‐ and post‐workshop respondent groups were not identical, the findings were examined in two complementary ways: a group‐level comparison of the proportion selecting high‐confidence or agreement responses, and an exploratory within‐participant comparison among the 12 respondents who completed both surveys (Table 2, Fig. 2).

**Table 2:** Group‐level and matched‐pair comparison of participant‐reported confidence and attitudes. Scores of 4–5 indicate higher confidence for confidence items and agreement for attitude items. All‐respondent data compare the pre‐workshop and post/follow‐up respondent groups descriptively. Matched‐respondent data show the direction of within‐participant change among the 12 respondents who completed both surveys. These 12 are 12 of the 13 respondents in the all‐respondent post/follow‐up column, so the two sets of columns describe almost the same individuals and do not corroborate one another independently. Higher, unchanged and lower scores indicate the direction of change on the response scale and should not automatically be interpreted as improvement or deterioration. For the academic‐integrity‐risk item, a lower score indicates reduced agreement that AI poses significant risks to academic integrity. The like‐for‐like comparison for the 12 matched respondents is shown in Fig. 2.

Survey item	All respondents – *n*/*N* (%)		Matched respondents – *n* (%)		
	Pre‐workshop (4–5)	Post/follow‐up (4–5)	Higher post score	Unchanged score	Lower post score
Confidence using AI for course design	12/21 (57.1)	12/13 (92.3)	7 (58.3)	4 (33.3)	1 (8.3)
Confidence using AI for teaching‐activity design	10/21 (47.6)	11/13 (84.6)	7 (58.3)	3 (25.0)	2 (16.7)
Confidence using AI for teaching‐material creation	9/21 (42.9)	10/13 (76.9)	8 (66.7)	2 (16.7)	2 (16.7)
Confidence in designing assessments that address AI use	8/20 (40.0)	12/13 (92.3)	6 (50.0)	4 (33.3)	2 (16.7)
Providing guidance for students on AI use	7/20 (35.0)	10/13 (76.9)	7 (58.3)	2 (16.7)	3 (25.0)
Belief that AI can enhance student learning	19/21 (90.5)	12/13 (92.3)	3 (25.0)	9 (75.0)	0 (0.0)
Belief that AI poses risks to academic integrity	11/21 (52.4)	2/13 (15.4)	1 (8.3)	5 (41.7)	6 (50.0)
Belief that AI will change assessment	17/21 (81.0)	11/13 (84.6)	7 (58.3)	3 (25.0)	2 (16.7)
AI as an opportunity rather than a threat	17/21 (81.0)	11/13 (84.6)	4 (33.3)	6 (50.0)	2 (16.7)

**Fig. 2:**
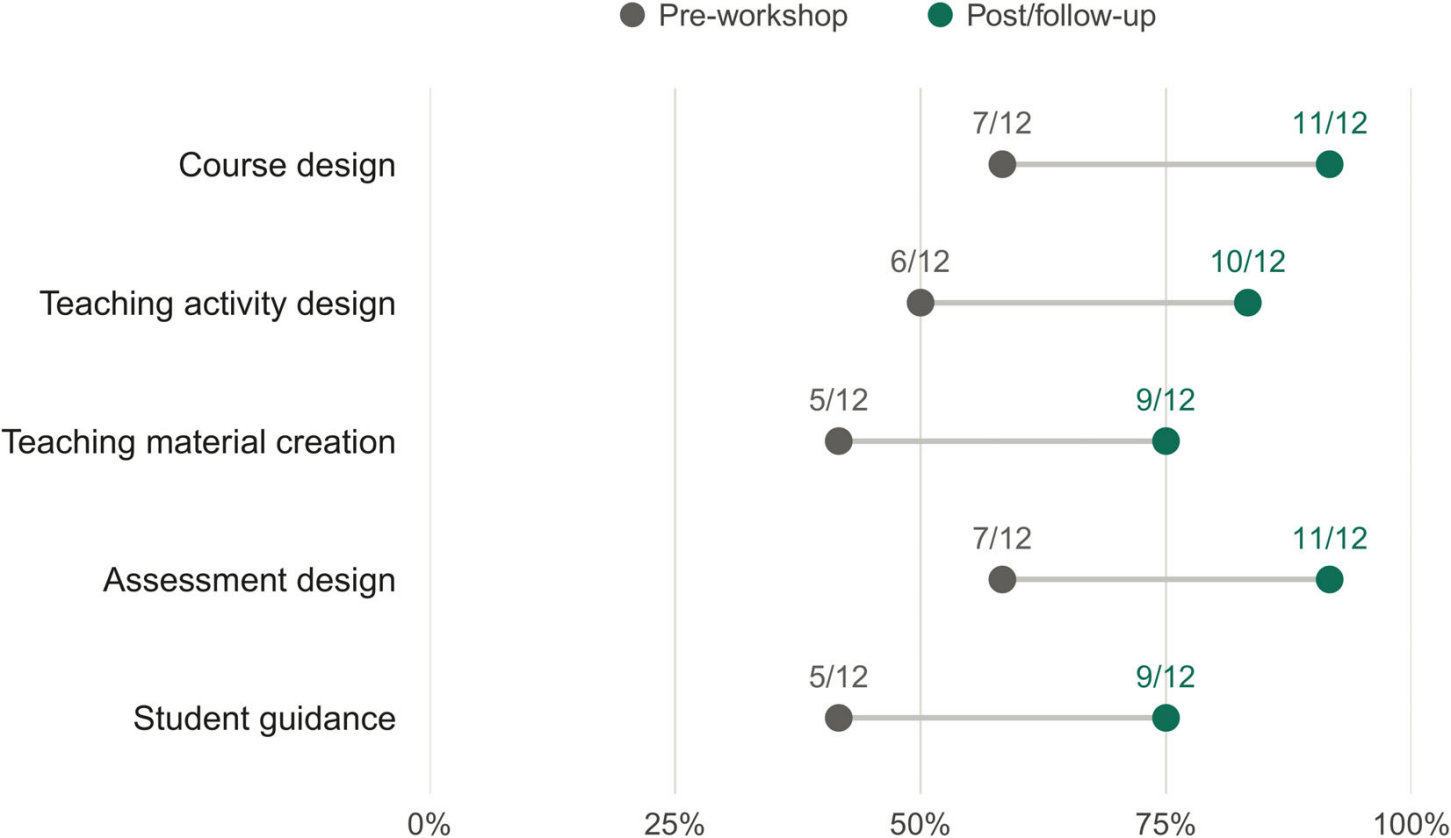
Participant‐reported confidence in practical AI‐use domains before and after the workshop among the 12 respondents who completed both surveys. Points show how many of these 12 respondents selected 4 or 5 on each 5‐point item before the workshop (grey) and at follow‐up (green). The student‐guidance item asks about current practice rather than confidence, and its wording differed between the two surveys (Doc S3). Group‐level proportions for all 21 pre‐workshop respondents (20 for assessment design and student guidance, each item having been left blank by one respondent) and all 13 follow‐up respondents are given in Table 2.

Overall, the two analyses showed a consistent pattern for the practical AI‐use domains. At the group level, the proportion of respondents selecting scores of 4 or 5 was higher in the post/follow‐up survey for all five confidence‐related items (Fig. 2). In the matched‐pair analysis, at least half of the matched respondents reported higher post‐workshop scores for course design, teaching‐activity design, teaching‐material creation, assessment design and student guidance. Among the 12 matched respondents, the proportion selecting 4 or 5 rose by the same margin, 4 of 12, in each of the five practical domains, from between 5 and 7 of 12 before the workshop to between 9 and 11 of 12 after it (Fig. 2). The largest group‐level increases were observed for assessment design (40.0% to 92.3%) and student guidance (35.0% to 76.9%). These larger differences largely reflect who completed the follow‐up survey rather than a large change within individuals. Pre‐workshop respondents who could not be matched to a follow‐up response had low baseline confidence in assessment design (1 of 8 selecting 4 or 5) and student guidance (2 of 8), whereas in the other three domains they resembled those who completed both surveys.

Attitude items showed a different pattern. Agreement that AI can enhance student learning, that AI will change assessment and that AI is an opportunity rather than a threat was already high before the workshop and remained high afterwards. By contrast, agreement that AI poses significant risks to academic integrity was lower in the post‐workshop respondent group than in the pre‐workshop group (11/21, 52.4%, versus 2/13, 15.4%), and half of the matched respondents recorded lower post‐workshop scores for this item. This is a fall in agreement on that specific response scale. It is not a favourable result and was not an aim of the workshop, and its interpretation is considered further in the Discussion.

Implementation data from the post/follow‐up survey indicated that workshop ideas were beginning to be applied by respondents. Ten of 13 respondents (76.9%) reported that they had implemented at least one idea from the workshop to some extent: seven selected ‘Yes’ and three selected ‘Yes, but not as much as I wanted to’. The remaining three respondents selected ‘Not yet, but planning to’, and no respondent selected ‘No’ (Table 3). These figures reflect self‐reported implementation by those who completed the follow‐up survey over a short follow‐up period and should be interpreted with the responder bias discussed below in mind. The threshold for this item was the implementation of at least one idea, which is a low bar, and the response options offered only one unqualified negative; both features would tend to raise the observed proportion.

**Table 3:** Self‐reported implementation of workshop ideas (*N* = 13).

Response option	*N* (%)
Yes	7 (53.8)
Yes, but not as much as I wanted to	3 (23.1)
Not yet, but planning to	3 (23.1)
No	0 (0.0)

### Expectations, planned changes and remaining challenges

Open‐text responses were analysed to clarify three points: what participants hoped to gain from the workshop, which session elements they identified as immediately applicable and where they still needed further support. The pre‐workshop survey included one open question on expected outcomes, and 17 of 21 respondents provided usable responses. The dominant expectation was practical support for integrating AI into teaching workflows, followed by course, activity or material design; assessment and academic integrity; and responsible use (Table 4). This suggests that participants entered the workshop looking primarily for applied educational support rather than a general introduction to AI. These expectations broadly matched the workshop scope, which covered practical AI use, course and activity design, teaching materials, assessment, academic integrity and responsible use.

**Table 4:** Pre‐workshop expectations (*N* = 17). Responses were coded thematically and could receive more than one code; therefore, counts do not sum to the number of usable responses. Percentages are calculated using the 17 usable pre‐workshop expectation responses as the denominator. This table describes expectation themes and does not represent a measure of expectation fulfilment or satisfaction.

Expectation theme	Example content captured	*N* (%)
Practical AI integration and tool use	Practical tips: how, when and why to use AI; integration into teaching workflows	11 (64.7)
Course, teaching activity or material design	Course design, learning designs, teaching activities, student engagement or educational materials	8 (47.1)
Assessment, academic integrity or regulation	Assessment redesign, student assignments, regulation of AI use or academic integrity	6 (35.3)
Ethical or responsible use	Ethical use, transparency, maintaining learning quality or critical thinking	5 (29.4)
Confidence, tool selection or understanding AI capabilities	Gaining confidence, selecting appropriate tools or understanding AI capabilities	4 (23.5)
Peer learning or international comparison	Learning how colleagues or institutions in other countries approach AI use	2 (11.8)
Student engagement or active student use	Enhancing engagement or integrating active student use of AI	2 (11.8)

The planned‐change responses from the one‐minute papers were broadly consistent with the focus of each session. After Session 1, participants mainly identified changes related to course outcomes, syllabus structure and alignment; after Session 2, changes related to teaching materials, visuals, active learning and AI‐supported workflows; and after Session 3, changes related to AI‐aware assessment, rubrics and assessment redesign. These responses indicate that participants were able to identify immediately applicable changes within each session. Because the planned changes closely reflected the session topics, these data were used descriptively rather than presented as a separate table.

The remaining‐uncertainty responses were more informative for identifying where participants may need additional scaffolding in future workshops (Table 5). After Session 1, uncertainties most often concerned risks, ethical constraints, institutional policy and the PICRAT framework. The prominence of PICRAT as a remaining uncertainty suggests that pedagogical frameworks may require more explanation, concrete examples, or guided application when participants are disciplinary academics with varying levels of formal training in educational design. After Session 2, the main challenges were prompting and quality control, suggesting that participants recognised AI‐supported material and activity design as an iterative process requiring judgement, checking and refinement rather than simple tool use. After Session 3, the most frequent single response was that no remaining concern was reported (5/18, 27.8%); among the substantive responses, concerns related to fairness, equity, privacy, academic integrity and institutional acceptance, which are issues that are not fully addressable through individual lecturer practice alone and that point to the need for transparent student guidance and institutional‐level support. Session 3 responses were, however, coded into nine categories against five for each of Sessions 1 and 2, so the apparent redistribution of concerns across the workshop sequence may reflect coding granularity rather than a measured shift.

**Table 5:** Remaining uncertainties and challenges reported after each workshop session. Responses to the remaining‐uncertainty question in each one‐minute paper were coded thematically. Themes were not mutually exclusive; therefore, counts may exceed the number of responses for each session. Percentages use the number of responses for the relevant session as the denominator: Session 1, *N* = 17; Session 2, *N* = 16; Session 3, *N* = 18.

Session and focus	Example content captured	*N* (%)
Session 1: foundations and course design	Risks, ethical constraints, institutional policy or uploading materials to AI tools	6 (35.3)
	PICRAT framework or its practical application	6 (35.3)
	Constructive alignment or programme‐level alignment	1 (5.9)
	No remaining uncertainty reported	3 (17.6)
	Vague or missing response	2 (11.8)
Session 2: activity and material design	Prompting	6 (37.5)
	Quality control, accuracy, depth or repetitive AI outputs	5 (31.3)
	Student engagement	4 (25.0)
	Access to premium or paid AI tools	2 (12.5)
	No remaining challenge reported	1 (6.3)
Session 3: academic integrity and assessment	Equity, access or paid/free tool differences	3 (16.7)
	Academic integrity, ethical use or fair use of AI in assessment/theses	3 (16.7)
	Privacy, fairness or consistency of AI‐supported evaluation	2 (11.1)
	Student agency or critical evaluation of AI outputs	2 (11.1)
	Alignment, alternative assessment or evaluating competencies	2 (11.1)
	Hallucination	1 (5.6)
	Institutional acceptance or governance	1 (5.6)
	Facilitator use or personalised feedback	1 (5.6)
	No concern, more comfortable, unclear or missing response	5 (27.8)

Overall, the open‐text responses suggest that the workshop addressed a practical need for AI‐related educational support while also identifying areas that require additional attention in future iterations: clearer scaffolding of pedagogical frameworks, more practice with prompting and quality control, and stronger guidance on fair, transparent and institutionally supported AI use in assessment.

### Follow‐up implementation

The online follow‐up meeting was attended by four participants and two facilitators, and this limited attendance should be weighed against everything reported from it below. The post/follow‐up survey had asked respondents whether they planned to attend: nine of 13 respondents (69.2%) selected either ‘Yes, I plan to attend’ or ‘Probably yes’, three (23.1%) selected ‘Not sure yet’, and one (7.7%) selected ‘Probably not’. Despite the small number of attendees, the discussion provided examples of early implementation. Participants described using AI tools for course restructuring, alignment with learning outcomes, lecture preparation, slide generation, translation of teaching materials and assessment redesign. Several examples involved iterative or multi‐tool workflows, and participants emphasised the need to adapt AI‐generated outputs to fit the level, scope and feasibility of their own courses. The discussion also identified practical barriers. Participants reported difficulties with style consistency across AI‐generated slide sets, limited editability of image‐based outputs, insufficient scientific depth when generating lectures from minimal prompts and the need for manual checking of AI‐supported translations. Scientific visuals were a particular concern, with examples including inaccurate chemical structures, anatomical errors, distorted workflow diagrams and garbled text in generated figures. Student use of AI was also discussed. Participants noted that AI tools may help students produce more polished presentations but raised concerns that students may not critically evaluate AI‐generated visuals or may not disclose AI use. Overall, the follow‐up discussion suggested that participants had begun to use AI in course design, teaching‐material preparation and assessment design, while also identifying continuing support needs related to quality control, student AI literacy, transparency and tool use.

## Discussion

This evaluation suggests that a short, hands‐on and context‐sensitive faculty‐development workshop can be a useful way to support life‐science educators in responding to generative AI. The value of the workshop lay not simply in introducing AI tools, but in giving participants structured time to work with their own teaching materials, examine possible uses and limitations, and discuss practical concerns with colleagues. This matters because AI integration in education is not a purely technical problem: it requires pedagogical judgement, disciplinary expertise, institutional awareness and continuing reflection. The findings are consistent with our initial premise that a bring‐your‐own‐materials, pedagogy‐first design would raise confidence in the practical domains it addressed, although the evaluation was not designed to test that intention. Within the 12 participants who completed both surveys, the proportion reporting high confidence rose by the same margin in all five practical domains. The larger group‐level differences for assessment design and student guidance, the areas in which participants entered least confident (Table 2), are explained by who completed the follow‐up survey rather than by a larger effect of the workshop in those domains. We interpret these results as evidence of feasibility and perceived usefulness rather than measured competence. Three considerations constrain how far they can be taken. The group‐level and within‐participant analyses share 12 of 13 respondents, so their agreement is arithmetic rather than independent corroboration; no within‐participant change reached significance on an exact sign test; and self‐reported confidence is known to correlate poorly with observed competence [21]. These data should therefore not be read as evidence that participants became more skilled, only that they felt more able to proceed. One result warrants particular caution. Agreement that AI poses significant risks to academic integrity was much lower in the post‐workshop respondent group than in the pre‐workshop group (11/21, 52.4%, versus 2/13, 15.4%), and was the only item for which most matched respondents moved downward. The simplest explanation is a demand effect. Session 3 was explicitly designed to move educators away from prohibition and detection framings; participants reported their views to the facilitators who had just taught them, on a name‐linked form; and the item itself asked about agreement that AI poses ‘significant risks to academic integrity’, wording that may have been read as endorsing the prohibition stance the session had argued against. On that reading the item records exposure to the workshop's framing rather than a change in judgement. A second reading is better calibration, with participants leaving with a more accurate sense that AI‐aware assessment design, rather than prohibition or detection, is the more workable response, and therefore feeling less alarmed. Third is over‐reassurance, in which a single positively framed workshop reduces vigilance about genuine integrity risks that remain unresolved at the institutional level. Our data cannot distinguish these interpretations, and fairness, equity, privacy and integrity concerns were still being raised after Session 3 (Table 5), even though a substantial minority of respondents recorded no remaining concern at that point. We state plainly that a fall in integrity concern is not an aim of this or any comparable workshop and should not be counted as a positive outcome. Separating three readings requires evidence that does not depend on self‐report. A future iteration should collect the assessment tasks participants actually redesign, together with their written rationale for which tasks permit disclosed AI use and which do not, and should have those artefacts rated against defensible‐assessment criteria by someone outside the facilitator team. Whether reported integrity concern rises or falls is then secondary to whether the assessments participants produce are ones they can defend. The baseline association between greater teaching experience and lower AI use, although exploratory and based on heavily tied ordinal data, is also worth noting. If more experienced educators are slower to adopt AI for teaching, disciplinary faculty development may need to target this group specifically. PICRAT was itself among the most frequent residual uncertainties after Session 1, although the Session 1 question had itself named PICRAT as an example (Doc S4), so that frequency should not be over‐read. The session materials nonetheless suggest why it may have remained unclear. The framework was presented in the foundation briefing and again after a live demonstration on a Biochemistry I syllabus, but the demonstration showed prompting for syllabus review rather than the mapping of the resulting changes onto the framework, so participants without formal training in educational design met PICRAT as a concept to apply rather than a tool they had seen used. In a re‐run we would invert that order and have participants map one of their own assignments onto the Replace‐Amplify‐Transform axis before the conceptual briefing, so that the framework is introduced to explain something they have already attempted rather than as an abstraction to be applied afterwards. For example, by foregrounding time‐saving, low‐risk applications and by ensuring that frameworks and prompting are scaffolded for colleagues with less formal training in educational design, who in our data found PICRAT a frequent source of residual uncertainty. More broadly, the findings support the importance of localised training. Educators work within different curricula, assessment cultures, institutional policies, student populations and resource constraints. Generic AI guidance can raise awareness but is unlikely to be sufficient for meaningful change in teaching and assessment practice. Workshops that allow educators to apply AI‐related ideas to their own courses may help bridge the gap between general principles and usable educational practice. This is consistent with broader faculty‐development literature emphasising active, practice‐based and context‐relevant professional learning [11], with competency frameworks for teachers' AI use [13], and with analyses of generative‐AI professional‐development needs [14]. The direction of our confidence findings is consistent with that reported for a TPACK‐framed workshop series in medical education [15] and for an introductory generative‐AI course for higher‐education staff [16], although all three evaluations share a reliance on self‐report and on small, self‐selected samples. What the present design adds is disciplinary specificity and the requirement that participants work on their own materials throughout rather than on supplied examples. FEBS Education and Training Academy is well positioned to support this kind of development in the molecular life sciences. As a disciplinary educational network, it can provide more than one‐off training: it can create spaces where educators compare approaches, share examples, discuss unsuccessful attempts and adapt emerging practices to the needs of life‐science education. This is particularly important for AI, where tools change quickly and where disciplinary accuracy, scientific representation, assessment validity and student responsibility are central concerns. A key implication is that AI‐related faculty development should be understood as a continuing process rather than a single intervention: workshops can help educators begin experimenting in structured and pedagogically informed ways, but sustained progress will require follow‐up opportunities, local discussion groups, shared resources and communities of practice [12].

This evaluation has clear limitations. The sample was small and drawn from educators who chose to attend an AI workshop, and whose attitudes toward AI were already positive at baseline (Table 2). Even as description, the findings extend only to comparable groups, and not to educators who are more sceptical about AI, who work with less institutional support or less reliable access to tools, or who teach in settings not represented among the 14 countries attending. Survey and follow‐up participation were incomplete (21/34 pre‐workshop, 13/34 post‐workshop, 4 at the follow‐up meeting), and all outcomes were self‐reported using an instrument developed for this evaluation rather than a validated scale. The surveys were also name‐linked and were administered by the workshop facilitators, who are the authors of this paper: participants reported their confidence to the people who had just taught them, in a form those people could attribute to them individually. Social‐desirability and demand effects are therefore a plausible contributor to the direction of every participant‐reported result presented here [20], and they cannot be separated from any genuine effect of the workshop. Importantly, those who found the workshop most useful and who acted on it are also the most likely to have completed the follow‐up survey and attended the meeting. This responder (and attrition) bias would tend to inflate the apparent gains in confidence and the reported implementation rate, and the partly overlapping samples across the four data sources further limit comparability. On the qualitative side, the responses were coded by the same individuals who delivered the workshop, and Session 3 was coded at a finer granularity than Sessions 1 and 2, which limits comparison across the sequence. In addition, each session's remaining‐difficulty question offered examples of what might still be unclear (Doc S4): PICRAT, risks and prompting after Session 1, and prompting, quality control and student engagement after Session 2. Several of those examples reappear among the most frequent themes reported for the same sessions, so the questions may have shaped the answers they collected, and the thematic frequencies in Table 5 should be read with that in mind. Participants came from 14 countries with widely differing institutional policies, but country‐level information was not analysed, and participants worked in six groups whose members are likely to have influenced one another's stated plans. The findings should therefore be interpreted as descriptive evidence of feasibility, perceived usefulness and early implementation, not as evidence of causal impact. Future work could use larger and more representative cohorts, comparison conditions, longer follow‐up, and assessment of the quality of redesigned teaching and assessment materials, as well as how participants influence local institutional practice after returning to their own contexts.

## Conclusions

A short, hands‐on, pedagogy‐first workshop built around participants' own teaching materials offers a transferable model for helping life‐science educators integrate generative AI into course design, teaching and assessment. In this evaluation, the 12 participants who completed both surveys reported increased confidence by the same margin in all five practical domains; 10 of the 13 follow‐up respondents reported implementing at least one idea, while attitudes toward AI as beneficial were already positive and changed little. Because the evidence is participant‐reported and uncontrolled, the main contribution of such workshops may be less about teaching specific tools and more about building the confidence, judgement and community that educators need for responsible AI integration. The workshop design, prompting framework and evaluation approach described here can be re‐run and adapted by FEBS Education and Training Academy and comparable disciplinary networks, ideally embedded in continuing communities of practice rather than delivered as isolated events.

## Conflict of interest

The authors designed, delivered, and facilitated the workshop being evaluated. They also administered the surveys, held the name‐linked response file, and coded qualitative data. The authors therefore had a direct interest in a favourable evaluation, and no independent party was involved in data collection or analysis.

## Author contributions

ZM and ABÖ conceived and designed the workshop and the evaluation. ZM and ABÖ facilitated the workshop. ZM and ABÖ collected and curated the data. ZM and ABÖ performed formal analysis. ZM and ABÖ drafted, reviewed, and edited the manuscript.

## Supporting information


**Doc S1**. Workshop specification.
**Doc S2**. Prompt library.
**Doc S3**. Pre‐ and post‐workshop survey instruments.
**Doc S4**. Qualitative data‐collection prompts.

## Data Availability

The complete session plans (Doc S1), prompt library (Doc S2), pre‐ and post‐workshop survey instruments (Doc S3) and one‐minute paper and follow‐up meeting questions (Doc S4) are provided as Supporting Information, so that the workshop can be re‐run without contacting the authors. The anonymised, aggregated data supporting the findings of this study are available from the corresponding author upon reasonable request. Individual‐level survey responses cannot be shared because participants were not consented for public release of raw data and because the small, multi‐national cohort poses a re‐identification risk.
