# Supplementary material for: Design and evaluation of FEBS Education and Training Academy module – Integrating generative artificial intelligence into life‐science education
Source: FEBS Open Bio. 2026 Oct 26:10.1002/2211-5463.70359. Online ahead of print. doi: 10.1002/2211-5463.70359 (PMC13699949; doi:10.1002/2211-5463.70359)
Supplement: Supplementary file 1 [file FEB4-9999-0-s001.pdf]

Supporting Information

Design and evaluation of FEBS Education and Training Academy module – Integrating generative artificial intelligence into life-science education

Zvonimir Mlinarić [1], Ali Burak Özkaya [2]

[1] Department of Pharmaceutical Analysis, Faculty of Pharmacy and Biochemistry, University of Zagreb, Croatia

[2] Faculty of Medicine, İzmir University of Economics, Türkiye

Zvonimir Mlinarić and Ali Burak Özkaya contributed equally to this article.

Correspondence:

Z. Mlinarić, Department of Pharmaceutical Analysis, Faculty of Pharmacy and Biochemistry, University of Zagreb, Ante Kovačića 1, 10000 Zagreb, Croatia
Tel: +38513060655
E-mail: zvonimir.mlinaric@pharma.unizg.hr

These documents are provided so that the workshop can be re-run and adapted without contacting the authors. They may be reused and adapted with attribution.

Doc S1. Workshop specification and session plans

Doc S2. Prompt library used in the workshop

Doc S3. Pre- and post-workshop survey instruments

Doc S4. Qualitative data-collection prompts: one-minute papers and the follow-up meeting

Doc S1. Workshop specification and session plans

Overview

- **Title:** Integrating AI into Education: Enhancing Course Design, Teaching, and Assessment
- **Setting:** a FEBS Education and Training Academy activity delivered at FEBS ETC 2026, Kuşadası, Türkiye, 26 to 28 March 2026
- **Format:** three face-to-face sessions of 2 h each, one per day, followed by a post-workshop follow-up component. FEBS Education and Training Academy workshops are planned as 9-h modules; the 6 h delivered face to face at FEBS ETC 2026 were completed by the online follow-up component
- **Participants:** academics and course coordinators involved in life-science education; planned for 30, attended by 34 from 14 countries, working in six groups
- **Mode:** in person, laptop-based; each participant uses a generative AI tool of their own choice
- **Facilitators:** two

Aim

To use generative AI tools to enhance learning through a pedagogically informed approach.

Learning goals

At the end of the workshop, participants will be able to:

- Describe the main functions, benefits and limitations of generative AI tools relevant to teaching and learning;
- identify ethical, privacy-related and bias-related considerations when using AI in educational settings;
- apply basic prompting techniques to obtain useful feedback or suggestions for course design and teaching materials;
- modify existing course elements, such as outcomes, activities, materials or assessments, based on AI-generated feedback and their own judgement;
- outline simple, transparent guidance for appropriate AI use within their own courses.

Approach

Each participant works on one of their own courses throughout the workshop, using AI tools to analyse, receive feedback on and improve real materials such as syllabi, slides, activities and assessments. Every session follows the same pattern: a short interactive briefing with concepts and examples, a live demonstration, hands-on studio work on the participant's own materials, and discussion. Participants are not asked to accept AI outputs directly; the emphasis throughout is on the educator's judgement, quality control, disciplinary accuracy and alignment with intended learning goals.

The PICRAT framework (Kimmons, Graham and West, 2020) is the recurring pedagogical anchor. Both axes are introduced in Session 1: the students' relationship to the technology (Passive, Interactive, Creative) and the educator's aim in using it (Replace, Amplify, Transform). The educator's axis is revisited in every subsequent briefing, and the students' axis returns in Session 3 in the context of academic integrity.

Before the workshop

On 18 March 2026, eight days before the workshop, registered participants received an e-mail invitation containing the link to the pre-workshop survey (Doc S3) and instructions on what to bring. The full text is reproduced at the end of this document. In summary, participants were asked to bring a laptop with access to a generative AI tool with sufficient usage capacity, and one of their own teaching materials for each session:

- **Session 1:** a course syllabus, or a document containing course outcomes and a weekly plan;
- **Session 2:** a lecture presentation, or a document describing the lecture design and learning objectives;
- **Session 3:** an assignment including its rubric, or a set of assessment questions.

Basic familiarity with AI tools was stated to be sufficient.

Session 1. Foundations and course design (2 h)

Part 1. Foundations

- **Foundation briefing (about 15 min).** Aims and plan of the workshop. What generative AI does: large language models as probabilistic output generators that generate, transform and interact. Prompting and the four-element prompt

structure (Doc S2, section 1). Concerns: dataset bias, hallucination, data security and the digital divide. Tools in common use: ChatGPT, Copilot, Gemini and Claude. The PICRAT framework, both axes.

- **Group discussion (about 20 min).** Why do faculty use AI less, or less effectively, than students? Each group identifies one major challenge and one strategy that would realistically reduce the gap, ideally one that has already worked. A representative of each group reports back.

Part 2. Course design

- **Short briefing (about 10 min).** How course design can be improved with generative AI, considering PICRAT. The elements of a syllabus: course aim and outcomes, the list of educational activities, laboratory practicals and assessment strategies.
- **Live demonstration (15 to 30 min).** A third-year Biochemistry I syllabus is reviewed with a generative AI tool for the clarity and quality of its outcomes, the alignment between outcomes and weekly content, the appropriateness of its assessment strategies, internal inconsistencies and missing elements (Doc S2, section 2). The Replace-Amplify-Transform axis of PICRAT is then shown again before the studio task.
- **Studio (30 to 45 min).** Participants rework their own syllabus or course plan with AI feedback, personalising the analysis to their own details (number of students, teaching assistants, infrastructure, student feedback), focus (learning outcomes, arrangement, coverage, practicals, assessments) and restrictions (programme requirements, rules and regulations, accreditation, core competencies).
- **One-minute paper** (Doc S4).

Intended outcome: an AI-informed understanding of ethical use and an improved course structure.

Session 2. Activity and material design (2 h)

Part 1. Activity design

- **Short briefing (10 to 15 min).** An educational activity as a structured learning experience designed to achieve specific learning goals within time and space restrictions. How generative AI can analyse slides, activity plans or other

documents, help align an activity with its goals and the course outcomes, and suggest alternative formats and engagement strategies, with an emphasis on active learning. PICRAT, educator's axis.

- **Live demonstration (15 to 30 min).** Slides for a carbohydrates session for first-year medical students are reviewed for alignment with the learning goals and for clarity, and then redesigned into a timed 1-h session with at least one active-learning component (Doc S2, section 3.1).
- **Studio.** Participants generate and refine one classroom activity from their own materials, personalising to their focus (learning goals, active learning, time management, aligning, merging or dividing activities, practicals, student input) and restrictions (experience with active learning, time and space).

Part 2. Material design

- **Short briefing (about 15 min).** Learning materials as the resources that support learning before, during or after an activity: slides, cases, figures and graphs, summaries and study notes, questions and examples. How generative AI can create materials, offer alternatives, suggest visuals, and give feedback on density, fonts and colours, on level, difficulty and complexity, and on goal alignment. PICRAT, educator's axis.
- **Demonstrations.** Generating a figure and an illustration, study notes and questions, a think-pair-share activity and a short clinical case; prompts for analysing and transforming existing material (Doc S2, section 3.2).
- **Short briefing (about 15 min).** How generative AI can support students' self-paced learning: generating their own learning materials, asking questions at any time, receiving explanations adapted to their level, AI as an on-demand tutor, and AI as a guide through Gibbs' reflective cycle (Doc S2, section 3.3).
- **Studio (about 30 min).** Participants obtain AI feedback on their own materials and improve them.
- **One-minute paper** (Doc S4).

Intended outcome: a redesigned, AI-assisted classroom activity and refined supporting materials.

Session 3. Academic Integrity and assessment design (2 h)

Part 1. Academic integrity

- **Short briefing (about 15 min).** Evidence on how students use AI in assessment. PICRAT, students' axis (Passive, Interactive, Creative). Why policing and banning do not work: detection tools lack consistency and accuracy, are biased against non-native English writers, produce high rates of false positives and unfair accusations, and are outpaced by bypass tools, while students and faculty overestimate their own ability to detect AI. Roadmaps that map AI functions onto each stage of a task, stating where students should use AI, how and why, and where they should avoid it and why, with a worked essay roadmap (Doc S2, section 4.2). Focusing assessment on process rather than output. Designing AI-aware rather than AI-proof assessment. AI literacy: prompt well, expect errors, revise critically, and build a conversation history.
- **Group discussion (about 30 min).** How does AI affect assessment fairness and transparency, and what can be done at the level of the lecturer and of the institution?

Part 2. Assessment design

- **Short briefing (about 10 min).** Assessment as part of the learning journey, meant to guide thinking, improve understanding and provide feedback rather than only to evaluate. PICRAT, educator's axis. How generative AI can analyse assessments and give feedback on clarity, difficulty, alignment and inclusivity; transform formats, for example from multiple-choice to case to short answer; recycle previous questions; and generate or improve rubrics and feedback. What to provide when creating or grading assessments with AI, and why its output should not be used directly.
- **Live demonstration (about 20 min).** Analysing an exam, a final assessment, multiple-choice questions, a rubric and an assignment; building an AI-aware assessment roadmap; an AI-aware case simulation; converting a short-answer question into a multiple-choice question; generating a rubric; designing an AI-aware assessment; proposing alternative assessment formats; creating a case-based assignment; and provisional grading against a rubric (Doc S2, section 4).

- **Studio (about 30 min).** Participants first analyse one of their own assignments, assessments, rubrics or questions, and then create an assessment, aiming to make it AI-aware.
- **One-minute paper** (Doc S4).
- **Announcement** of the follow-up component.

Intended outcome: a revised, ethically sound assessment plan and a course-level AI-use statement.

Follow-up component

- **Post-workshop survey** (Doc S3): invitation sent on 18 April 2026, which also announced the date of the follow-up meeting, with reminders on 1 May and 1 June 2026.
- **Online follow-up meeting,** 6 June 2026, 11:00 CEST, Microsoft Teams: a semi-structured, discussion-based session on participants' experiences since the workshop, organised around four questions circulated with the meeting details on 1 June 2026 (Doc S4).

Pre-workshop invitation e-mail, 18 March 2026

> Dear Participants,
> We are very pleased to welcome you to the FEBS Education and Training Academy Workshop "Integrating AI into Education: Enhancing Course Design, Teaching, and Assessment", taking place at FEBS ETC 2026.
> Over the course of three days, we will explore how artificial intelligence can meaningfully support and transform course design and curriculum development, teaching strategies and student engagement, assessment design and feedback practices, as well as ethical, responsible, and transparent AI integration.
> To ensure that the workshop is tailored to your needs, experiences, and expectations, we kindly ask you to complete a short pre-workshop survey about your current use of AI in education. Please complete the survey here: [survey link]. The survey will take only a few minutes to complete. Your responses will help us understand the diversity of AI experience in the group, identify key challenges and opportunities, and design hands-on activities that are relevant and practical. We would appreciate it if you could complete the survey by March 24th.

> What is expected from participants: as this will be a highly interactive and hands-on workshop, you will need a working laptop and access to a GenAI tool with sufficient usage capacity (e.g., OpenAI's ChatGPT, Microsoft Copilot, Google Gemini, Claude AI, or another platform you prefer). During the workshop, you will be working directly on your own educational materials. More specifically:
>
> > - Session 1: Please bring either a course syllabus or a document containing course outcomes and a weekly plan.
> > - Session 2: Please bring a lecture presentation (slides) or a document describing the lecture design and learning outcomes.
> > - Session 3: Please bring either an assignment (including its rubric) or a set of assessment questions.
>
> You do not need to be an expert user of AI tools. Basic familiarity is completely sufficient. The goal is to work directly with your own teaching materials and experiment with practical applications in a supportive environment.
>
> If you have any specific expectations, questions, or topics you would especially like us to address during the workshop, please feel free to reply to this e-mail. We very much look forward to meeting you and working together on exploring thoughtful and effective ways to integrate AI into education.
>
> With best regards,
>
> Ali Burak Özkaya and Zvonimir Mlinarić, Workshop Facilitators, FEBS Education and Training Academy, FEBS ETC 2026

Post-workshop survey invitation, 18 April 2026

> Dear Participants,
>
> We hope you have been well since the workshop at FEBS ETC 2026.
>
> It has now been a few weeks since we worked together on rethinking course design, activities, materials, and assessments with AI. Many of you shared concrete ideas and plans during the sessions, and we are very curious to see how these have evolved in your own contexts.
>
> To better understand what has been implemented so far, and what challenges you may have encountered, we would greatly appreciate it if you could complete this short follow-up survey: [survey link].

> The survey will take approximately 3–5 minutes to complete. Your responses will help us shape the next steps and ensure that the follow-up session is as relevant and useful as possible.
> We are also pleased to confirm that our online follow-up meeting will take place on June 6th, 11:00 CEST. We will use this session to share experiences, discuss what has worked (and what has not), and continue building on the ideas developed during the workshop.
> As we experienced during the workshop, this process is not always straightforward, but it becomes much more meaningful when done together.
> Thank you once again for your engagement, openness, and willingness to question and improve your own practice.
> We look forward to hearing from you and seeing you again in June.
> With best regards,
> Ali Burak Özkaya and Zvonimir Mlinarić, Workshop Facilitators, FEBS Education and Training Academy, FEBS ETC 2026

Follow-up meeting details, 1 June 2026

> Dear Friends,
> As our online follow-up session approaches, we would like to share a few details about the format of the meeting.
> The session will be discussion-based and will focus on your experiences since the workshop. We would like to hear from each participant and invite everyone to contribute to the conversation. To guide the discussion, we will ask participants to reflect on the following questions:
>
> > - What have you implemented, and how did it go?
> > - What challenges did you encounter?
> > - What additional support, knowledge, or resources would have helped?
> > - What will you try next?
>
> We strongly encourage open discussion, sharing of experiences, and offering advice to one another. One of the strengths of the workshop was learning from colleagues facing similar opportunities and challenges, and we hope to continue that spirit during the follow-up session.
> Meeting details: 6 June 2026, 11:00 CEST, [Microsoft Teams link].
> We would also like to take this opportunity to remind those who have not yet completed the follow-up survey to do so if possible: [survey link].

> Thank you again for your engagement and willingness to share your experiences. We look forward to seeing you on 6 June.
>
> With best regards,
>
> Ali Burak Özkaya and Zvonimir Mlinarić, Workshop Facilitators, FEBS Education and Training Academy, FEBS ETC 2026

Doc S2. Prompt library used in the workshop

Prompts are reproduced as they were used in the workshop demonstrations, with typographical corrections only. Words in square brackets are placeholders to be replaced with the user's own content. In the demonstrations the examples were drawn from first-year medical biochemistry and a third-year biochemistry course; in the studio sessions participants adapted the prompts to their own courses, students and institutional constraints.

1. The four-element prompt structure

Prompting was taught as a design skill under the principle "rubbish in, rubbish out". Every prompt should specify four elements:

- **Context:** where is this used?
- **Task:** what exactly should the AI do?
- **Constraints:** what rules should it follow?
- **Output:** what should the result look like?

The same request, underspecified and then specified:

> Generate a question about metabolism.
>
> Generate a multiple-answer question on the regulation of the Krebs cycle for first-year medical students in a biochemistry exam. Students have not yet covered glycolysis.

Two further habits were demonstrated. First, the AI can be asked to help write the prompt:

> Create a prompt for [task]... but before responding, ask me five questions to clarify [what you need to know].

Second, iterate within the same conversation, so that the tool retains the context already given, rather than starting again. Participants were also reminded to consider what they can legally and ethically upload.

2. Session 1. Course design

2.1 Reviewing a syllabus (live demonstration)

> Please review and revise the syllabus of a Biochemistry I course. This is a 3rd-year chemistry course, following organic chemistry and preceding Biochemistry II (focused on metabolism).
>
> Analyse the syllabus for:
>
>> - clarity and quality of course outcomes;
>> - alignment between outcomes and weekly content;
>> - whether all weekly content maps to one or more outcomes;
>> - appropriateness of assessment strategies for the outcomes;
>> - internal inconsistencies in the document;
>> - missing elements compared to similar courses.
>
> Then:
>
>> - suggest specific improvements;
>> - revise poorly written outcomes;
>> - highlight misalignments clearly.

In the studio, participants added their own details, focus and restrictions (Doc S1, Session 1) to the same structure.

3. Session 2. Activity and material design

3.1 Redesigning a teaching activity (live demonstration)

Step 1, review:

> Review my slides for a carbohydrates session for first-year medical students.
>
> Context:
>
>> - 30 students, no teaching assistant;
>> - small classroom with movable chairs;
>> - learning goals are included in the slides.
>
> Tasks:
>
>> - evaluate alignment between slides and learning goals;
>> - identify issues with clarity or structure;
>> - suggest improvements.

Step 2, redesign, in the same conversation:

> Then redesign this into a 1-hour session with:
>
>> - timed segments;

> > - at least one active learning component (e.g., live polling, think-pair-share, peer instruction);
> > - clear alignment between goals and activities.
>
> For each segment, include:
>
> > - instructor role;
> > - student activity.

A single-step alternative:

> Review my slides for a carbohydrates session for first-year medical students.
>
> Context:
>
> > - 30 students, no teaching assistant;
> > - small classroom with movable chairs;
> > - 1-hour educational activity.
>
> Tasks:
>
> > - suggest timed segments;
> > - suggest at least one active learning component (e.g., live polling, think-pair-share, peer instruction) to be included.

3.2 Creating and improving learning materials

A figure. Shown first underspecified, as "Create a clear visual showing the fate of pyruvate after glycolysis for first-year medical students.", and then specified:

> Create a clear visual showing the fate of pyruvate after glycolysis for first-year medical students.
>
> Requirements:
>
> > - pyruvate as the central starting point;
> > - a clear split into aerobic and anaerobic pathways;
> > - aerobic: acetyl-CoA → TCA cycle;
> > - anaerobic: lactate formation;
> > - keep it simple and uncluttered.
>
> Output: a single image.

The generated figures were used to discuss the accuracy of AI-generated scientific visuals, which must always be checked.

An illustration.

> Create an illustration for a first-year medical biochemistry lecture showing the four major macromolecules as comic-style superheroes: carbohydrates, lipids, proteins, and nucleic acids.
>
> Requirements:
>
> > - each macromolecule is a distinct character inspired by its structure and function;
> > - keep the style clean and simple and not childish;
> > - avoid clutter and excessive text;
> > - use subtle labels (names and key function only).
>
> Output: a single cohesive scene with all four characters.

Study notes and questions.

> Based on the uploaded slides, create study notes and questions for 1st-year medical school students.
>
> Tasks:
>
> > - summarise key concepts into clear, structured notes;
> > - highlight essential mechanisms and relationships.
>
> Then generate:
>
> > - 3–5 concept-check questions;
> > - 2–3 application or clinical questions.
>
> Output:
>
> > - organised notes (bullet points or short sections);
> > - questions with answers and brief explanations.
>
> Ensure alignment with the learning goals in the slides.

An active-learning activity.

> Design a think-pair-share activity for first-year medical students on the reason for lactate production under anaerobic conditions.
>
> Requirements:
>
> > - use one multiple-choice question that tests conceptual understanding and reasoning (not recall);
> > - include plausible distractors based on common misconceptions;
> > - keep the activity simple, feasible and under 10 minutes.
>
> Output:
>
> > - the single discussion question;
> > - brief instructor instructions.

A clinical case.

> Create a short clinical case for first-year medical students related to carbohydrate metabolism (e.g., glycolysis, pyruvate metabolism, or lactate production).
>
> Requirements:
>
> > - ensure it connects basic biochemistry to a clinical context;
> > - avoid unnecessary medical complexity.
>
> Then generate 3–4 clinically relevant questions that require reasoning and application (not recall).
>
> Output:
>
> > - the clinical case;
> > - the questions;
> > - correct answers with brief explanations.

Analysing and transforming existing material. Follow-up prompts, each specifying the dimension to be changed (learning objectives or difficulty, year or level, time restrictions, topic):

> Analyse the case for [dimension]...
>
> Simplify the case...
>
> Transform this case to...
>
> Write a similar case, but...

3.3 Prompts for student's self-paced learning

These were presented as examples that educators can give to students.

Generating their own study material from a document:

> Create 10 exam-style questions based on this document.
>
> Generate flashcards for the key concepts.
>
> Explain the main argument in simpler terms.
>
> Give me a short quiz to test my understanding.

Using AI as an on-demand tutor:

> Explain this concept as if I am a beginner.
>
> Give me a real-world example.
>
> Ask me questions to test my understanding.
>
> Explain the difference between these two concepts.

A guided reflection tutor using Gibbs' reflective cycle. The complete prompt, which students paste at the start of a conversation:

> You are a structured reflection facilitator. Your role is to guide me through Gibbs' Reflective Cycle step by step. Follow these rules strictly.
> General rules:
>
>> - Follow Gibbs' Reflective Cycle exactly in this order: Description, Feelings, Evaluation, Analysis, Conclusion, Action Plan.
>> - Ask only one question at a time.
>> - Do not move to the next stage until I answer the current question.
>> - If my answer is vague or incomplete, ask a short follow-up question before moving forward.
>> - Do not skip steps, summarise early, or introduce any other reflection frameworks.
>> - Keep your questions clear, concise, and focused on reflection.
>> - At the end of the cycle, produce one structured reflection document that includes each question you asked and my response, organised according to the six stages of Gibbs' cycle.
>
> Step 1, Description. Ask: "What happened? Describe the experience or situation in detail. Focus on the facts: where it happened, who was involved, and what occurred." Wait for my response before continuing.
> Step 2, Feelings. Ask: "What were you thinking and feeling during the experience?" Wait for my response before continuing.
> Step 3, Evaluation. Ask: "What aspects of the experience went well, and what aspects did not go well?" Wait for my response before continuing.
> Step 4, Analysis. Ask: "Why do you think the situation unfolded the way it did? What factors or underlying causes influenced what happened?" Wait for my response before continuing.
> Step 5, Conclusion. Ask: "What have you learned from this experience? What could you have done differently?" Wait for my response before continuing.
> Step 6, Action Plan. Ask: "If a similar situation happens again, what will you do? What specific steps will you take to improve your response or performance?" Wait for my response before continuing.
> Final output. After all six stages are completed, generate one structured reflection document in the following format: "Gibbs' Reflective Cycle Summary", then for each of the six stages in order (1. Description, 2. Feelings, 3. Evaluation, 4. Analysis, 5. Conclusion, 6. Action Plan) the question asked and my response. Do not add commentary beyond organising the reflection.

4. Session 3. Academic integrity and assessment design

4.1 Analysing existing assessments

An exam, against the course materials.

> Review the attached midterm exam alongside the provided course materials (lecture slides, learning objectives, and study notes) for first-year medical school students.
>
> Tasks:
>
>> - evaluate alignment with the learning objectives;
>> - assess coverage of key concepts from the materials;
>> - determine if the level and difficulty are appropriate;
>> - identify unclear, ambiguous, or misleading questions;
>> - review for inclusivity, ensuring it accommodates diverse learners (including neurodivergent students) in terms of accessibility and cognitive load.
>
> Then suggest specific improvements. Keep feedback concise and practical.

A final assessment, against the syllabus.

> Review the attached final assessment alongside the course syllabus for first-year students.
>
> Tasks:
>
>> - evaluate alignment between the assessment and learning outcomes;
>> - assess coverage of key topics from the syllabus;
>> - determine if the level and difficulty are appropriate;
>> - identify gaps, redundancies, or over- or under-representation of topics;
>> - review for inclusivity, ensuring it accommodates diverse learners (including neurodivergent students) in terms of accessibility and cognitive load.
>
> Then:
>
>> - suggest specific improvements;
>> - recommend changes to better align the assessment with the course.
>
> Keep feedback concise and practical.

Multiple-choice questions.

> Analyse the attached MCQs and learning objectives prepared for a medical biochemistry exam for first-year medical students.
>
> Check:
>
>> - clarity and ambiguity;
>> - alignment with the attached learning objectives.
>
> Then:
>
>> - suggest improvements if needed;

> • provide a brief explanation of why the correct answer is correct and why the others are incorrect.

A rubric.

> Review the attached grading rubric, instructions and learning objectives for a first-year medical course essay.
>
> Tasks:
>
> - evaluate alignment with the learning objectives;
> - assess clarity and usability for first-year students;
> - identify gaps, ambiguities, or inconsistencies.
>
> Then:
>
> - suggest specific improvements;
> - revise unclear or weak criteria if needed.
>
> Keep feedback concise and practical.

An assignment.

> Review the attached assignment instructions and learning objectives for first-year students.
>
> Tasks:
>
> - evaluate the clarity of the assignment instructions;
> - assess whether the time required is appropriate;
> - determine if the level matches the students;
> - check alignment with the learning objectives;
> - review for inclusivity, ensuring it accommodates diverse learners (including neurodivergent students) in terms of accessibility and cognitive load.
>
> Then:
>
> - identify any issues or ambiguities;
> - suggest specific improvements.
>
> Keep feedback concise and practical.

4.2 Making an assessment AI-aware

An AI-aware assessment roadmap for students.

> You are redesigning an existing assessment to be AI-aware. Based on the provided assessment task, learning objectives, and student level and context, create a clear, student-facing roadmap that guides how to approach the task using AI in a way that supports learning.

> Task breakdown: divide the assessment into meaningful stages (e.g., understanding, planning, drafting, refining).
> AI use by stage: for each stage, specify how AI can be used and how AI should not be used.
> Rationale for learning: briefly explain why this use of AI supports learning at each stage.
> Checkpoints and milestones: define moments where students must demonstrate their own thinking (e.g., in-class discussion, draft submission, reflection).
> Transparency requirement: specify how students should document their AI use (e.g., prompts, outputs, reflections).
> Final output expectation: clarify what must reflect the student's own understanding and what is AI-supported work.
> Keep the roadmap clear and structured, actionable for students, and focused on learning, not restriction.

Example roadmap presented in the briefing, for an essay.

> 1. Understand the task. Use AI to clarify the question, identify key concepts and plan the process. Avoid producing the essay immediately.
> 2. Initial research. Use AI to analyse resources and as a repository. Avoid treating AI output as a source.
> 3. Draft writing. Use AI to get feedback on clarity and to ask for examples or explanations. Avoid generating full paragraphs and submitting them.
> 4. Refinement and argumentation. Use AI to challenge your argument and identify gaps or weaknesses.
> 5. Editing and polishing. Use AI to improve grammar and clarity.

Example of a process-focused, AI-aware assessment presented in the briefing.

> In-class activity: introduction, understanding the task and planning the research with generative AI, with prompts provided and AI use supervised.
> Assignment 1, initial research supported by generative AI: students submit the full conversation with the AI and the sources the AI used.
> Assignment 2, writing the essay: manual writing followed by generative AI polishing; students submit both their own version and the AI-polished version.

Designing a new AI-aware assessment.

> Design an AI-aware assessment for first-year students based on the attached learning objectives and course context.
> Requirements:
>
> > - focus on process over final product;

> - include in-class or supervised components;
> - break the task into milestones (instead of a single submission);
> - require students to use AI in a controlled or transparent way (e.g., in-class use or submission of AI conversations).
>
> Then:
>
> - provide a clear assessment structure (steps and timeline);
> - create an AI usage roadmap (what to use AI for at each step);
> - briefly explain how this design supports learning and integrity.
>
> Keep it practical and feasible for a typical class setting.

Proposing alternative assessment formats.

> Based on the attached assessment, learning objectives, and course context (number of students, level, and constraints), propose alternative assessment formats.
>
> Tasks:
>
> - suggest 2–3 alternative assessment types;
> - ensure alignment with the learning objectives;
> - consider feasibility (e.g., class size, time, resources);
> - review for inclusivity, ensuring it accommodates diverse learners (including neurodivergent students) in terms of accessibility and cognitive load.
>
> For each alternative:
>
> - briefly describe the format;
> - explain how it improves or complements the original assessment.

Keep suggestions practical and appropriate for first-year students.

4.3 An AI-aware case simulation

Presented as an answer to the question of how a case-solving assessment can be made AI-aware and process-focused. The student works through the case with the AI, and the transcript, rather than the final answer alone, is assessed.

> You are now a medical case simulation for a first-year medical student. The full case is contained in the uploaded file. Do not reveal the full case at once and do not allow the student to simply peek at the answer. Your role is to reveal information gradually, only in response to the student's questions, as if they are working through a real case.
>
> Rules:
>
> - only use information contained in the file;
> - do not invent new findings unless asked to clarify something already present;

> - do not immediately confirm the diagnosis or mechanism unless the student has gathered enough evidence;
> - encourage the student to explain their reasoning;
> - if the student asks for a test or history detail, provide the relevant information only if it is available in the case;
> - if the student jumps too quickly to a conclusion, ask them what evidence supports it.

> When the student states their final answer, generate a complete transcript of the interaction in the following format: "Case Simulation Transcript. Step 1. Student: ... AI: ... Step 2. Student: ... AI: ...", including all exchanges in chronological order. Keep it clean and exportable (plain text, no extra commentary). This transcript will be used for evaluation.

4.4 Creating assessment items and rubrics

Converting a short-answer question into a multiple-choice question.

> Convert the attached short-answer question into a multiple-choice question (MCQ) for first-year medical students.
> Learning objective: "Explains the reason for lactate production after glycolysis in anaerobic conditions."
> Indicate the correct answer and provide a brief explanation of why the correct answer is correct and why the others are incorrect.

A case-based assignment.

> Create a case-based assignment for first-year medical students.
> Learning objective: "Explains the reason for lactate production after glycolysis in anaerobic conditions."
> Include:
> - a short, realistic clinical case;
> - 2–3 questions requiring reasoning and application;
> - brief model answers or key points.
>
> Keep it clear, relevant, and appropriate for first-year level.

A rubric.

> Create a grading rubric for the attached assignment instructions based on the attached learning objectives for first-year medical students.
> Include:
> - clear criteria;
> - performance levels (e.g., excellent to poor);

> - brief descriptors for each level.

4.5 Provisional grading

> Grade the attached student essay for first-year medical students using the attached rubric and example grading.
>
> Tasks:
>
> - assign a provisional score for each criterion;
> - provide brief, constructive feedback;
> - highlight strengths and areas for improvement.

The briefing set out what to provide when creating assessments with AI (learning objectives, lecture slides, study notes or key concepts, and the level of the students) and when grading with it (the rubric or marking scheme, model answers or key points, learning objectives, key concepts and the level of the students). Participants were told not to use the output directly: generative AI is a probabilistic output generator, hallucinates, is biased, and when grading, may expose student work to a third party.

Doc S3. Pre- and post-workshop survey instruments

Both surveys were administered online in English using Google Forms and are reproduced here verbatim, including their titles and introductory text. Every item was required except the final comment question of the post-workshop survey. Respondents entered their first and last name, which was used to match pre- and post-workshop responses. Agreement items were preceded by the instruction quoted below; on the response scale itself only the end points were labelled.

Neither form contained a consent statement or any reference to research use. Consent to the use of anonymised, aggregated evaluation data for research and publication, and to the linking of pre- and post-workshop responses by name, was obtained orally at the start of the workshop (see the Methods of the main article).

Pre-workshop survey

Title: "FEBS Education and Training Academy Workshop Integrating AI into Education: Enhancing Course Design, Teaching, and Assessment Survey"

Open from 18 March 2026 until the start of the workshop on 26 March 2026. No introductory text.

1. First and Last Name (short answer)

2. What is your primary role?

Professor · Lecturer · Educational developer · Teaching assistant · Other (free text)

3. Years of teaching experience

0-5 · 6-10 · 11-20 · 20+

4. Have you used AI tools (e.g., OpenAI's ChatGPT, Microsoft Copilot, Google Gemini, Claude AI) for teaching-related tasks?

Regularly · Occasionally · Tried once · Aware, but not used · Not familiar

5. How often do you use AI for teaching-related tasks?

Never · Monthly · Weekly · Several times per week · Daily

6. Have you modified your teaching or assessment practices because of AI?

Yes, significantly · Yes, slightly · No, but I plan to · No

Instruction preceding items 7 to 17: "Please rate the extent to which you agree with the following statements using the following scale: 1 – Strongly disagree, 2 – Disagree, 3 – Neither agree nor disagree, 4 – Agree, 5 – Strongly agree"

Each of items 7 to 17 was a linear scale from 1 to 5, with the end points labelled "Strongly disagree" (1) and "Strongly agree" (5).

- 7. I feel confident using AI tools to support course design (e.g., creating or refining learning outcomes, syllabus structure, or weekly topics).
- 8. I feel confident using AI tools to design teaching activities (e.g., lectures, discussions, or active learning exercises).
- 9. I feel confident using AI tools to create teaching materials (e.g., slides, examples, visuals, or explanatory content).
- 10. I feel confident designing assessments that appropriately address AI use.
- 11. I provide a clear guidance on whether and how AI tools may be used in this course.

- 12. I feel confident critically evaluating AI-generated outputs for their accuracy, quality, and suitability for educational use.
- 13. I believe AI can enhance student learning when used appropriately.
- 14. I believe AI poses significant risks to academic integrity.
- 15. I believe AI will fundamentally change how we assess students.
- 16. I see AI as an opportunity rather than a threat in education.
- 17. My institution provides sufficient guidance on AI use.

18. What is one specific outcome you hope to gain from this workshop? (short answer)

Post-workshop survey

Title: "FEBS Education and Training Academy Workshop Integrating AI into Education: Enhancing Course Design, Teaching, and Assessment Follow Up Survey"

Introductory text: "This short follow-up survey aims to understand how participants have engaged with and implemented ideas from the AI in Education workshop in their teaching practice. Your responses will help us evaluate the impact of the workshop and shape future support and sessions. The survey takes approximately 3–5 minutes to complete."

The invitation was sent on 18 April 2026, with reminders on 1 May and 1 June 2026.

1. First and Last Name (short answer)

Instruction preceding items 2 to 10: identical to the pre-workshop survey. Each item was a linear scale from 1 to 5, with the end points labelled "Strongly disagree" (1) and "Strongly agree" (5).

- 2. I feel confident using AI tools to support course design (e.g., creating or refining learning outcomes, syllabus structure, or weekly topics).
- 3. I feel confident using AI tools to design teaching activities (e.g., lectures, discussions, or active learning exercises).
- 4. I feel confident using AI tools to create teaching materials (e.g., slides, examples, visuals, or explanatory content).
- 5. I feel confident designing assessments that appropriately address AI use.
- 6. I provide a clear guidance for students on whether and how AI tools may be used in courses I teach.
- 7. I believe AI can enhance student learning when used appropriately.
- 8. I believe AI poses significant risks to academic integrity.

- 9. I believe AI will fundamentally change how we assess students.
- 10. I see AI as an opportunity rather than a threat in education.

Section: FEBS Education and Training Academy WS Implementation and Follow Up

11. Have you implemented at least one idea from the workshop in your teaching?

Yes · Yes, but not as much as I wanted to · Not yet, but planning to · No

12. Are you planning to attend the follow-up session on June 6th, 11:00 CEST?

Yes, I plan to attend · Probably yes · Not sure yet · Probably not · No, I do not plan to attend

Respondents answering "Not sure yet", "Probably not" or "No, I do not plan to attend" were routed to item 13; all others went directly to item 14.

Section: Reason for not attending

13. If you are not planning to attend or not sure, what is the main reason?

Scheduling conflict · Not relevant to my needs · Other (free text)

Section: General Feedback

14. Anything else you want to share with us? (short answer, optional)

Correspondence between the two surveys

- **Repeated unchanged:** pre-workshop items 7 to 10 are post-workshop items 2 to 5, and pre-workshop items 13 to 16 are post-workshop items 7 to 10.
- **Repeated with changed wording:** pre-workshop item 11 ("I provide a clear guidance on whether and how AI tools may be used in this course") and post-workshop item 6 ("I provide a clear guidance for students on whether and how AI tools may be used in courses I teach"). The later version adds "for students" and widens the referent from the course the participant brought to the workshop to all the courses they teach. This item describes current practice rather than confidence.
- **Pre-workshop only:** items 1 to 6, item 12 (critically evaluating AI-generated outputs), item 17 (institutional guidance) and item 18.
- **Post-workshop only:** items 11 to 14.

Doc S4. Qualitative data-collection prompts

One-minute papers

Completed anonymously at the end of each face-to-face session using Google Forms, with no name field. Both questions were required and answered as free text. Each session asked for one intended change and one remaining difficulty, worded to match that session's topic.

Session 1. Foundations and course design (form title "Session 1 - One-minute activity")

- What is one change you plan to make to your course design (e.g., outcomes, structure, or alignment) based on today's session?
- What aspect of using AI for course design or ethical considerations (e.g., PICRAT, risks, prompting) is still unclear or difficult?

Session 2. Activity and material design (form title "Session 2 - One-minute activity")

- What is one change you plan to make to a teaching activity or learning material using AI?
- What challenge do you still face in designing activities or materials with AI (e.g., prompting, quality control, student engagement)?

Session 3. Academic integrity and assessment design (form title "Session 3 - One-minute activity")

- What is one change you plan to make to your assessment or academic integrity approach in response to AI?
- What concerns or difficulties remain about ensuring fair and effective assessment in an AI-supported context?

Online follow-up meeting

Held on 6 June 2026 at 11:00 CEST via Microsoft Teams as a semi-structured, discussion-based session. The four questions below were sent to all participants with the meeting details on 1 June 2026, and are quoted from that message (reproduced in full in Doc S1):

- What have you implemented, and how did it go?
- What challenges did you encounter?
- What additional support, knowledge, or resources would have helped?
- What will you try next?
